# An Efficient Ensemble Deep Learning Approach for Semantic Point Cloud Segmentation Based on 3D Geometric Features and Range Images

**DOI:** 10.3390/s22166210

**Published:** 2022-08-18

**Authors:** Muhammed Enes Atik, Zaide Duran

**Affiliations:** Department of Geomatics Engineering, Istanbul Technical University (ITU), Istanbul 34469, Turkey

**Keywords:** autonomous driving, deep learning, light detection and ranging (LiDAR), point cloud, semantic segmentation

## Abstract

Mobile light detection and ranging (LiDAR) sensor point clouds are used in many fields such as road network management, architecture and urban planning, and 3D High Definition (HD) city maps for autonomous vehicles. Semantic segmentation of mobile point clouds is critical for these tasks. In this study, we present a robust and effective deep learning-based point cloud semantic segmentation method. Semantic segmentation is applied to range images produced from point cloud with spherical projection. Irregular 3D mobile point clouds are transformed into regular form by projecting the clouds onto the plane to generate 2D representation of the point cloud. This representation is fed to the proposed network that produces semantic segmentation. The local geometric feature vector is calculated for each point. Optimum parameter experiments were also performed to obtain the best results for semantic segmentation. The proposed technique, called SegUNet3D, is an ensemble approach based on the combination of U-Net and SegNet algorithms. SegUNet3D algorithm has been compared with five different segmentation algorithms on two challenging datasets. SemanticPOSS dataset includes the urban area, whereas RELLIS-3D includes the off-road environment. As a result of the study, it was demonstrated that the proposed approach is superior to other methods in terms of mean Intersection over Union (mIoU) in both datasets. The proposed method was able to improve the mIoU metric by up to 15.9% in the SemanticPOSS dataset and up to 5.4% in the RELLIS-3D dataset.

## 1. Introduction

With the increasing usage of autonomous systems in vehicles, modeling, understanding, and interpretation of the environment becomes an important task. Robust and real-time sensing of the environment with high spatial accuracy is an important requirement for autonomous driving [1]. For this purpose, different sensors are used such as RGB camera, light detection and ranging (LiDAR), depth camera or Radar sensors. LiDARs are now a crucial part in perception systems due to direct space measurements, which provide accurate three-dimensional (3D) representation of the world [2]. Mobile point clouds obtained with LiDAR are used for many tasks such as object detection, object tracking, and semantic segmentation [3].

Mobile point clouds are data obtained using laser scanners mounted on a moving vehicle. The geometric information contained in point clouds is valuable as a basis for many applications. Accurate sense of environment and precise positioning are crucial requirements for reliable navigation and safe driving of autonomous vehicles in complex dynamic environments [4]. Mobile point clouds can be used in applications such as road network management, architecture and urban planning, and 3D high definition (HD) city maps for autonomous vehicles. For all these purposes, semantic segmentation of point clouds is an essential requirement [5].

Early on, traditional machine learning algorithms and rule-based methods were used for semantic point cloud segmentation [6]. However, due to the irregular data structure of the point cloud data, the complex geometric properties of the objects, and other noises, these methods are insufficient for accurate classification [7]. Recently, deep learning methods have been successfully applied for the segmentation of point clouds. Because mobile point clouds are large, complex, and irregular data, applying semantic segmentation is a challenging issue. Range image-based methods have been introduced to solve these issues. The irregular point cloud structure is projected on a 2-dimensional (2D) plane. Thus, the point cloud is transformed into a more organized and easier process.

**Figure 1:**
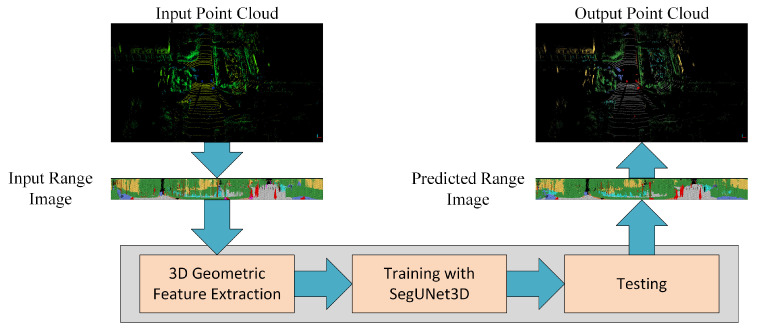
Workflow of the study.

In this study, we propose an ensemble point cloud semantic segmentation method that combines 3D data structure and 2D segmentation techniques. Range images are created by projecting the irregular structure of the point cloud to the 2D plane. Each point in the range image is defined by vectors containing geometric features. The proposed SegUNet3D approach is compared with other range image-based approaches, SqueezeSegv2, PointSeg, and SalsaNext. Additionally, SegUNet3D’s advantages over image-based SegNet and U-Net methods were demonstrated. Experiments are performed on mobile LiDAR datasets SemanticPOSS produced in an urban area and RELLIS-3D produced in a rural area. A search was also conducted for detecting optimum input and segment size for semantic segmentation. The workflow is presented in Figure 1.

## 2. Related Works

### 2.1. Semantic Point Cloud Segmentation with Point-Based Methods

PointNet [8] is the first method that is directly working on irregular point clouds. The fundamental idea behind PointNet is to learn each point’s spatial encoding and then gather all of them into a single global point cloud signature. Features are created with multi-layer perceptrons (MLPs) and clustered with the max-pooling function. A set of functions that select informative key points from a group of points and encode this information in each layer feature vector is learned by the network [9]. PointNet++ is based on two fundamental problems: setting the point set and isolating point sets or local features. PointNet++ uses a hierarchical neural network that employs PointNet recursively on the input point set [10]. Point sets are divided into local neighborhood areas that overlap based on a distance metric. The neighborhood radius is gradually increased and features are extracted. Small neighborhoods capture fine-grained local features, whereas large neighborhoods capture all shape geometry. PointNet ++ uses larger kernels to extract solid patterns from sparse point clouds. A random input dropout layer is introduced to learn a strategy optimized to combine multi-scale features. This layer randomly selects the input points according to a specific ratio from each sample.

The PointNet architecture pioneered the development of many methods for point cloud semantic segmentation. Reference [11] used a combination of K-clustering and KNN for defining two neighborhoods in world space and feature space separately. The proposed network has a structure where all points are passed through MLP and pooled in feature blocks by max pooling. RandLA-Net [12], which runs directly on point clouds, was developed for large-scale databases. It can process data quickly as it does not contain any preprocessing steps. RandLA-Net uses random point sampling to provide high efficiency in memory and computational cost. A local feature aggregation module is presented to capture complex local features and spatial relationships. PointCNN [13] learns an χ transformation from the input points, thereby weighting the points and preventing loss of shape information. Convolution is applied to χ-transformed points. Reference [14] was proposed as the PointWeb method to explore the relationship of all point pairs in a local neighborhood. Adaptive Feature Adjustment (AFA) module, a new module is available to find the interaction between points. An impact map is applied to the feature pairs map for each local region, which has an element-based effect between the point pairs. The features are well coded with region information and therefore take advantage of point cloud recognition tasks such as point cloud segmentation and classification. Another method is ShellNet [15] that is a permutation invariant convolution for point cloud deep learning. The ShellNet method defines representative features by using statistics from concentric spherical shells to solve point order ambiguity, and conventional convolution methods work on these features. MLPs and 1D CNNs are used to obtain the final output. Reference [16] present a convolution operator that is called Kernel Point Convolution (KPConv). KPConv is inspired by image-based convolution, but it uses kernel points to define the area where the kernel weight is applied instead of the kernel pixel used by image-based convolution. However, the location of the kernel points is also learned in the network, which allows the points to learn the topology of local neighborhoods and deform the voxel grid to suit such a topology. Reference [17] proposed a full convolutional neural network for airborne LiDAR point cloud classification. As the input data of the network are 3D coordinates and LiDAR intensity, the architecture can be applied directly to the point cloud. Another method was proposed by [18]. The proposed 1D convolutional neural network can perform point cloud segmentation not only with a point cloud but also with RGB obtained from a 2D geo-referenced image. The unclassified points are classified on the image by k-NN.

### 2.2. Semantic Point Cloud Segmentation with Voxel-Based Methods

Voxel-based methods use points to be grouped with regular shapes (cube, sphere, prism, etc.) as a basic unit instead of individual point. Early methods split the point cloud into voxels of certain sizes and 3D convolutions are applied to these voxels for semantic segmentation [5]. VoxNet [19] defined the points in the point cloud as a 3D binary occupancy grid. These occupancy grids are used as input to CNNs for semantic segmentation. High memory consumption is a crucial disadvantage of voxel-based methods, because of the unnecessary computation on empty voxels. Recently, the octree-structured voxelization has been widely used to hierarchically divide 3D point clouds to reduce memory consumption. The most critical parameter is the size of the local point group. There is a significant loss of information in the point cloud if the low voxel resolution is selected [20].

### 2.3. Semantic Point Cloud Segmentation with Projection-Based Methods

SqueezeSeg [21] proposes a new method for semantic point cloud segmentation that enables the evaluation of point clouds reorganized into a spherical range image. Thus, the semantic segmentation problem of point clouds is reduced to the image segmentation problem. The height of the point exists as a band of the image, even if it is not as a third dimension. Although SqueezeSeg was developed with the SqueezeNet architecture [22], it has 50 times fewer parameters. A down-sampled feature map is produced with the fireModules it uses. Then, convolution layers are used for upsampled and a probability map is produced. Produced probability maps are improved with a conditional random field (CRF). The same authors suggested the SqueezeSegv2 [1] method with some improvements. Context aggregation module (CAM) has been added to the SqueezeSeg architecture to eliminate the effect of dropout noise caused by missing points in the point cloud. Moreover, focal loss has been added due to class imbalance in the point clouds. Additionally, binary mask to LiDAR input data and batch normalization are added. PointSeg [23] uses the fire module from SqueezeNet for feature extraction. Multiple convolutions are applied with the enlargement layer to obtain more location information. A global average pooling layer is used to obtain the squeeze global information descriptor. RangeNet [24] is inspired by the Darknet53 architecture [25]. A 2D fully convolutional semantic segmentation is applied to the created range images. The estimated result images are transferred to the whole point cloud with a k-NN based approach. SalsaNext [26] is an enhanced version of SalsaNet, which is a encoder-decoder architecture. In SalsaNext, a residual dilated convolution stack with 1 × 1 and 3 × 3 kernels are added to the head of the network to improve context information. In addition, ResNet encoder blocks are replaced with a new residual dilated convolution stack. The pixel-shuffle layer is added in the decoder section. There are also studies [2] that apply image segmentation algorithms such as U-Net to the point cloud. A new point cloud segmentation approach was proposed by rearranging the PointNet++ architecture to be applied to range images in [3].

## 3. Materials and Methods

This section will present the recommended approach for LiDAR point cloud segmentation. The conversion of point clouds to range image, calculation of geometric features, and semantic segmentation stages through deep learning will be explained, respectively.

### 3.1. Datasets

The method proposed in the study was evaluated in two different datasets: SemanticPOSS and RELLIS-3D.

#### 3.1.1. RELLIS-3D

RELLIS-3D [27] is a large data set created to test semantic segmentation algorithms developed for robust and safe navigation of autonomous vehicles in off-road environments. Most of the data sets available in the literature present the urban environment, whereas RELLIS-3D, which provides off-road environment data, allows autonomous driving opportunities in different terrain structures. The dataset was collected from Texas A&M University’s Rellis Campus, including challenges to class imbalance and environmental topography. It contains 13,556 LiDAR scans and 6235 images of the off-road environment. The LiDAR scans are split as 7800/2413/3343 frames for our experiments. Its official release has 14 classes for LiDAR semantic segmentation: grass, tree, bush, concrete, mud, person, puddle, rubble, barrier, log, fence, vehicle, pole, and water.

#### 3.1.2. SemanticPOSS

The SemanticPOSS dataset [28] was obtained with the Hesai Pandora sensor module consisting of cameras and LiDAR sensors. Moreover, there is a GPS/IMU system for localization and orientation information. The point cloud was collected on a 1.5 km line at Peking University, China. SemanticPOSS contains 14 classes that are labeled similarly to the SemanticKITTI dataset. The semantic labels are unlabeled are: people, rider, car, trunk, plants, traffic sign, pole, trashcan, building, cone/stone, fence, bike, and road. Unlabeled points are ignored during the training and testing process. SemanticPOSS is divided into 6 parts, 500 frames per part. Part 3 is used for testing and the rest is for training.

### 3.2. Proposed Approach: SegUNet3D

#### 3.2.1. Producing Network Input: Range Images

**Figure 2:**
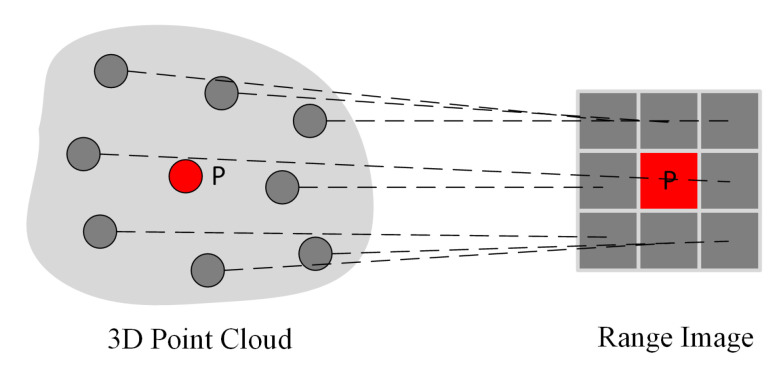
An illustration of the point cloud segment transformed to range image. Red point is center and gray points are neighbor points.

Point clouds contain Cartesian coordinates (*x*, *y*, *z*) as well as additional information such as RGB, intensity, and number of returns. Converting LiDAR point clouds to more compact structures simplifies the processing of point clouds and reduces the computational cost. A spherical projection method is proposed to convert irregular LiDAR point cloud structures into regular range images (Figure 2). The projection of the point cloud is realized with the intrinsic parameters of the LiDAR sensor. LiDAR point clouds are presented in a grid-based structure onto a sphere by calculating two parameters in Equations (1) and (2) [21].

(1) $$\theta =arcsin\frac{z}{\sqrt{x^{2}+y^{2}+z^{2}}},\tilde{\theta }=\left[\theta /▵\theta \right]$$

(2) $$\phi =arcsin\frac{y}{\sqrt{x^{2}+y^{2}}},\tilde{\phi }=\left[\phi /▵\phi \right]$$

**Figure 3:**
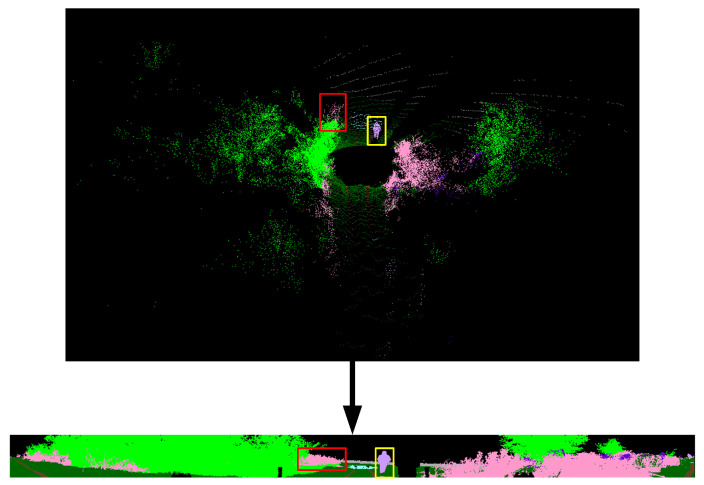
The captured point cloud data is projected to the 2D plane due to LiDAR parameters. Objects close to the sensor are denser, and the density decreases as you move away from the sensor. Some projected objects are marked with red and yellow rectangles.

θ and ϕ refer to azimuth and zenith angles, respectively. ▵θ and ▵ϕ are resolutions for discretization, $\tilde{\theta }$ and $\tilde{\phi }$ define 2D position of a point on the spherical grid. Range image provides a more structured, light and dense representation of the point cloud (Figure 3). Thus, it enables tasks that require fast data processing, such as autonomous driving, to be performed on lower hardware [21].

#### 3.2.2. Extraction of Geometric Features

Geometric features that can describe the local geometric properties of a point cloud are produced from the covariance matrix calculated from the local neighborhood area of the central point [29]. Let P=(x,y,z) be the central point. The points inside the sphere with the center point *P* are the neighboring points of *P*. A set of points with a certain Euclidean distance from other points is defined as a segment. Thus, the point cloud is segmented.

Covariance matrix is calculated for points within a segment. Covariance is a measure of how much each of the dimensions changes relative to the mean [30]. The covariance matrix of a segment centered at point P=(x,y,z) is:

(3) $$C=\left[\begin{array}{ccc} Cov(x_{i},\bar{x}) & Cov(x_{i},\bar{y}) & Cov(x_{i},\bar{z})\\ Cov(y_{i},\bar{x}) & Cov(y_{i},\bar{y}) & Cov(y_{i},\bar{z})\\ Cov(z_{i},\bar{x}) & Cov(z_{i},\bar{y}) & Cov(z_{i},\bar{z}) \end{array}\right]$$

where, *Cov(x, y)* is the covariance of *x, y* computed by using (4).

(4) $$Cov\left(x,y\right)=\frac{1}{n-1}\sum_{i=1}^{k}\left(x_{i}-\bar{x}\right)\left(y_{i}-\bar{y}\right)$$

where, *n* refers to afterwards, the eigenvalues of the covariance matrix *C* are calculated. The eigenvalues are ordered from largest to smallest as $\lambda_{1}$ > $\lambda_{2}$ > $\lambda_{3}$. After the eigenvalues are obtained, the geometric features are calculated. Eigen features calculated in this study are: *linearity* (5), *planarity* (6), *scattering* (7), *omnivariance* (8), *anisotropy* (9) *eigenentropy* (10), and *surface variation* (11) [31]. In addition to these seven features, height, intensity, range, normal angle are added to the feature vector.

(5) $$Linearity=\left(\lambda_{1}-\lambda_{2}\right)/\lambda_{1}$$

(6) $$Planarity=\left(\lambda_{2}-\lambda_{3}\right)/\lambda_{1}$$

(7) $$Scattering=\lambda_{3}/\lambda_{1}$$

(8) $$Omnivariance=\sqrt[{3}]{\lambda_{1}\lambda_{2}\lambda_{3}}$$

(9) $$Anisotropy=\left(\lambda_{1}-\lambda_{3}\right)/\lambda_{1}$$

(10) $$Eigenentropy=\sum_{i=1}^{3}\lambda_{i}\ln\lambda_{i}$$

(11) $$Surface\;variation=\lambda_{3}/\left(\lambda_{1}+\lambda_{2}+\lambda_{3}\right)$$

#### 3.2.3. Review of U-Net

Traditional CNN architectures contain sequential convolution layers and gain strong semantic attributes as they are deeper into the network architecture. In addition, while the pooling layers reduce the feature size, spatial details are lost. U-Net architecture is combined with interconnection layers and final layers to take more advantage of the spatial attributes in the first layers. In this way, it has benefited from the features obtained in the first layers, which are quite rich in terms of location information.

U-Net [32], which is one of the widely used perceptual algorithms in the segmentation of 2D data, contains multi-channel feature maps and has 23 convolution layers. It consists of parts, each containing 3 × 3 convolutions, a ReLU process, and 2 × 2 max-pooling layers. U-Net architecture, which has a symmetrical structure, enables one to spread the contextual information created in the feature layers to higher resolution layers. The general structure of the U-net architecture consists of a series of 2 × 2 subsampling layers followed by a 3 × 3 convolutional layer. In the convolution process, the activation function is used as the transformation function (Equation (12)).

(12) $$Z\left(x_{k}\left(ii,jj\right)\right)=f\left(\sum_{k=1}^{k}x_{k}\left(ii,jj\right)\times w_{k}+b_{k}\right)\Leftrightarrow Z=f\left(x\times w+b\right)$$

where *w* refers to weight vector, b is bias vector, and $x_{k}$(ii,jj) is the input of the activation function and the output of convolution operation [33].

#### 3.2.4. Review of SegNet

SegNet [34] is a fully convolutional neural network architecture with an encoder-decoder structure. The network consists of the encoder–decoder layers, convolution layers, batch normalization, pooling indices, and rectified linear unit (ReLU) parts. The first 13 layers of the encoder network correspond to the first 13 layers of the VGG-16 network. There is a corresponding decoder for each encoder. In each encoder and decoder network, several filters are applied to generate and normalize feature maps. The decoder generates sparse feature maps by upsampling the feature map using the memorized maxpooling indices from the corresponding encoder. Finally, class probabilities are calculated by using to Equation (13) to classify each pixel with softmax in the final decoder network.

(13) $$s\left(x_{i}\right)=\frac{e^{x}}{\sum_{j=1}^{n}e^{x}}$$

where *n* refers to the number of classes, *x* is the output vector of the model, and index *i* is in the range of 0 to n−1.

#### 3.2.5. Architecture

**Figure 4:**
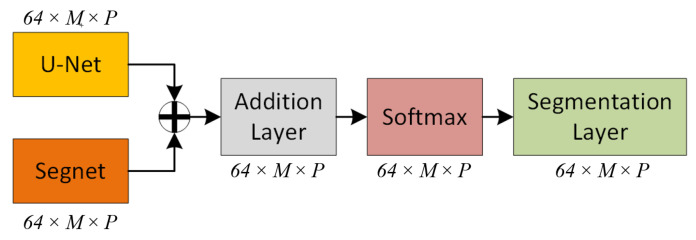
Addition of weights of two streams.

Each of the U-Net and SegNet architectures has been successful in inferring different classes in classification. Based on this finding, it was hypothesized that averaging the output weights of the algorithms would yield better results than the individual methods themselves. The created range images are processed in U-Net and SegNet architectures in two streams and weights are obtained in the designed architecture. The latest model weights are calculated by averaging the weights from the two channels. Two vectors of *64 × M × P*, *M* width size, and *P* weights from the last convolutions in decoder of U-Net and SegNet are summed. Then, the weights are normalized in the softmax layer and transferred to the segmentation layer (Figure 4).

**Figure 5:**
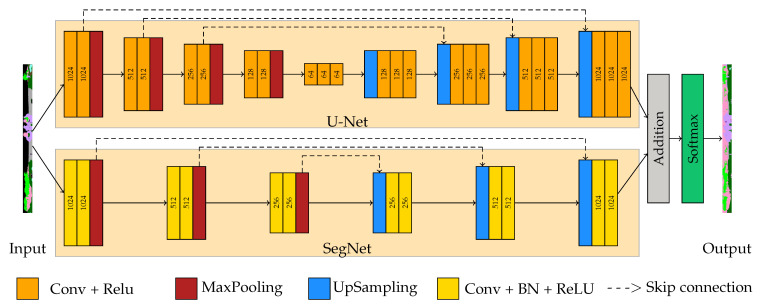
An illustration of a SegUnet3D architecture. The 64 × 1024 image is in two streams, downsampling in the encoder and then upsampling in the decoder. Thus, the input and output size will be the same. The specified numbers represent the width of the image in that layer.

The standard cross-entropy is used for the final semantic prediction of SegUNet3D. For each input element $y_{j}$, cross-entropy element-wise loss values are computed using Equation (14).

(14) $$Loss=-\frac{1}{N}\sum_{n=1}^{N}\sum_{i=1}^{K}w_{i}p_{i}^{n}log\left({\hat{p}}_{i}^{n}\right)$$

where *N* and *K* are the numbers of observations and classes, respectively. $w_{i}$ is weight for each i element, $p_{i}^{n}$ is 1 if pixel *i* labeled as *n*, ${\hat{p}}_{i}^{n}$ is predicted class probability [3]. The architecture of SegUNet3D is presented in Figure 5.

## 4. Results and Discussion

SegUnet3D and other methods were trained and evaluated on SemanticPOSS and RELLIS-3D datasets with the specified training parameters. Each sequence is initially organized in 64 × *N* (*N* is the width of the image) dimensions. Geometric features are calculated using reorganized point clouds, and range images are generated. The proposed SegUNet3D is compared with the range image-based SqueezeSegv2, PointSeg, and SalsaNext algorithms and image segmentation methods, U-Net and SegNet. In order to make a correct assessment, other methods were also trained and tested on the created data sets. All of the experiments are implemented in a MATLAB environment and performed with a single GPU. A total of 20 epochs, 0.9 momentum, and 0.001 initial learning rate were used as training parameters. Batch size is determined as 16 for 64 × 512 input size, 8 for 64 × 1024 input size, and 4 for 64 × 2048 input size, considering hardware capability. For the experiments, i7-11800H, 2.30 GHz processor, GTX 3070 graphics card, and 32 GB RAM hardware is used. The results are evaluated by the mean Intersection over-Union (mIoU).

(15) $$\mathrm{mIoU}=\frac{1}{N}\sum_{c=1}^{N}\frac{P_{c}\cap G_{c}}{P_{c}\cup G_{c}}$$

where $P_{c}$ and $G_{c}$, respectively, refer to predicted and ground-truth points that belong to class *c*. *c*∈ (1, 2, …, *N*) is the index of the class.

### 4.1. Comparative Experiment Analysis

Comparative experiment analyses were carried out on datasets to examine different experimental design. SegUnet3D and other methods are compared regarding with the effect of input sizes, segment size, and usage of 3D geometric features. Thus, the superiority of the SegUnet3D method over the methods in the literature was emphasized. The performance of the proposed algorithm in different terrain structures has been examined. Additionally, the performances of the methods on the basis of classes are also presented.

#### 4.1.1. Effect of Input Image Resolution

Firstly, the influence of the input image resolution explores the semantic point cloud segmentation. All of the architectures with 64 × 512, 64 × 1024, and 64 × 2048 input sizes were used for this study. According to the results, the effect of the input resolution size changes depending on the dataset. In the SemanticPOSS dataset, mIoU increases significantly when the input size is increased from 64 × 512 to 64 × 1024. However, although the mIoU increased from 64 × 1024 to 64 × 2048, 64 × 2048 has more inference time. Considering the effectiveness and efficiency, the experiments were performed for the SemanticPOSS dataset with 64 × 1024. The results are presented in Table 1.

For the RELLIS-3D dataset, the 64 × 512 input size is superior in terms of mIoU. There is an inverse relationship between input size and mIoU for SegUnet3D. However, SalsaNext has higher mIoU at 64 × 1024 and 64 × 2048, while SegUnet3D has a higher mIoU at 64 × 512. mIoU decreased from 64 × 512 to 64 × 2048. Therefore, 64 × 512 input size was preferred for RELLIS3D. The effect of input size on evaluation metrics in RELLIS-3D dataset is presented in Table 2.

#### 4.1.2. Effect of Segment Size

The point cloud is first divided into clusters for feature extraction. Points within a specified Euclidean distance are defined as a cluster. In this study, the distance was chosen as 0.5 m. The change of Euclidean distance does not significantly affect the number of clusters. The point number size of the cluster is the main parameter to be evaluated. As the minimum number of points decreases, the number of clusters increases. The SegUnet3D and other algorithms are tested according to the minimum number of points of 30, 50, and 70. In both data sets, the highest mIoU was reached in 50 points for SegUnet3D. Clusters consisting of 30 points remain small to define an object, whereas more than one object can remain in a cluster at 70 points. This has the effect of reducing mIoU. A total of 50 minimum points were determined as the optimum number of points and used in the experiments. The results for both datasets are shown in Table 3 and Table 4.

#### 4.1.3. Effect of 3D Geometric Features

Each of the calculated geometric features (Section 3.2.2) for a point is added to the range image as a band in addition to the 3D coordinates. As the input, images with the size of *h × w* × 10 pixels (*h, w*; height and width of image) with geometric features and images with the size of *h × w* × 3 pixels without geometric features were created. Thus, a point is defined using 3D coordinates and its local geometric features. More input features are provided to train the model. Points belonging to the same class are expected to have similar geometric features. In order to reveal the effect of geometric features, the SegUNet3D and other algorithms were applied to both data sets by changing only the feature space. According to the results, when 3D geometric features are added, mIoU metrics is increased. In the SemanticPOSS dataset, when results of SegUnet3D is examined, this increase was about 3.3% (Table 5), while in RELLIS-3D there was an increase of 6.7% in mIoU (Table 6). An increase in the mIoU value was also observed in other methods, except for U-Net.

### 4.2. Comparison with State-of-the-Art Models

The proposed approach is compared with three range image-based methods (SqueezeSegV2, PointSeg, and SalsaNext) and two image-based methods (U-Net and SegNet). All methods have been trained and tested using range images created by adding geometric features. Since the SemanticPOSS data set was created in the urban area, it includes more regular structures. RELLIS-3D usually contains more natural and irregular structures. SegUnet3D algorithm is superior to other methods in rural areas as well as in urban areas.

**Figure 6:**
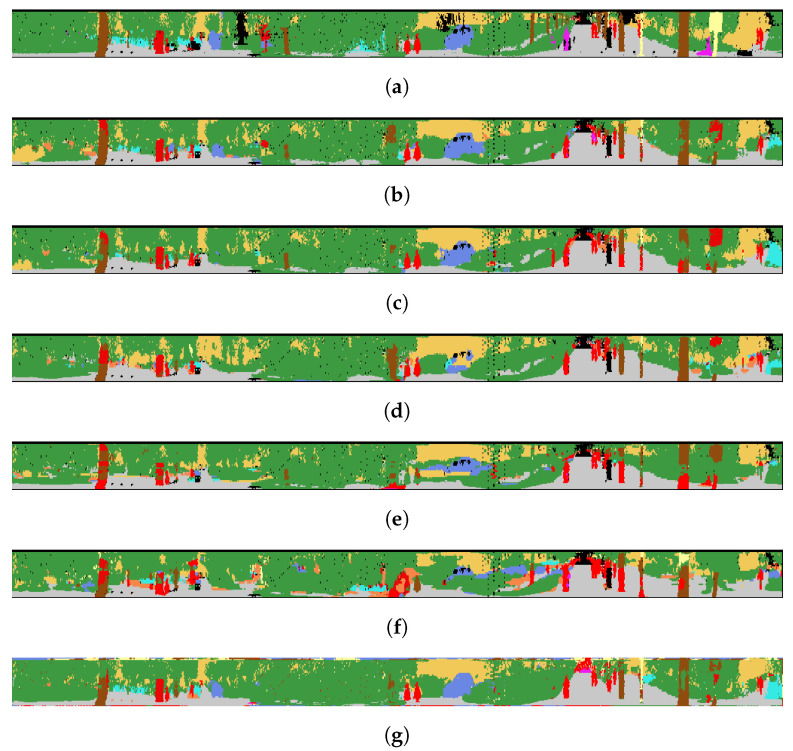
Qualitative results of the methods for SemanticPOSS. (**a**) Ground Truth; (**b**) SegUNet3D; (**c**) SegNet; (**d**) U-Net; (**e**) SqueezeSegV2; (**f**) PointSeg; (**g**) SalsaNext.

**Figure 7:**
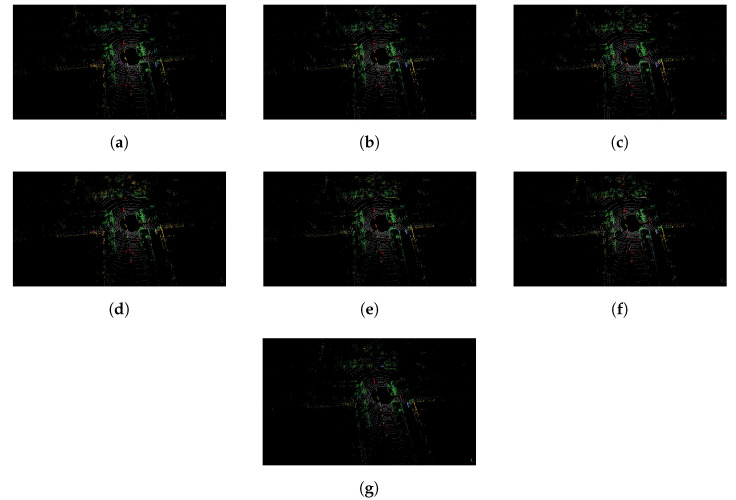
Semantic segmentation results of the SemanticPOSS dataset are presented as point clouds. (**a**) Ground Truth; (**b**) SegUNet3D; (**c**) SegNet; (**d**) U-Net; (**e**) SqueezeSegV2; (**f**) PointSeg; (**g**) SalsaNext.

The evaluation results of the SemanticPOSS dataset are shown in Table 7. For SemanticPOSS, the SegUNet3D method is significantly superior to image-based and range image-based methods. In particular, 9.2% higher mIoU value was obtained from SqueezeSegV2, and was 15.9% higher than PointSeg and 0.9% higher than SalsaNext. Especially in the building, road, and plant classes in the urban area, higher IoU value is obtained. However, lower IoU values were obtained in classes such as the rider, pole, and traffic sign. The main reason is the amount of training data. Building, road, and plant classes have higher evaluation metrics in semantic segmentation because the urban area has many buildings, roads, and plants. The semantic segmentation results of SemanticPOSS dataset are shown in Figure 6 and Figure 7.

**Figure 8:**
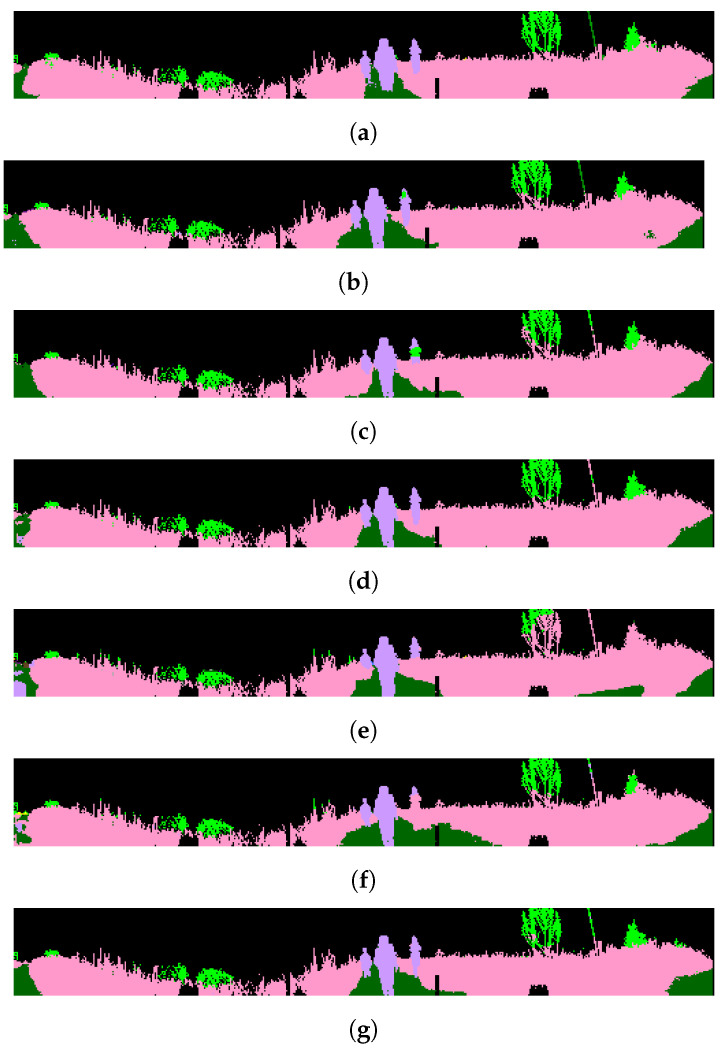
Qualitative results of the methods for RELLLIS-3D. (**a**) Ground Truth; (**b**) SegUNet3D; (**c**) SegNet; (**d**) U-Net; (**e**) SqueezeSegV2; (**f**) PointSeg; (**g**) SalsaNext.

**Figure 9:**
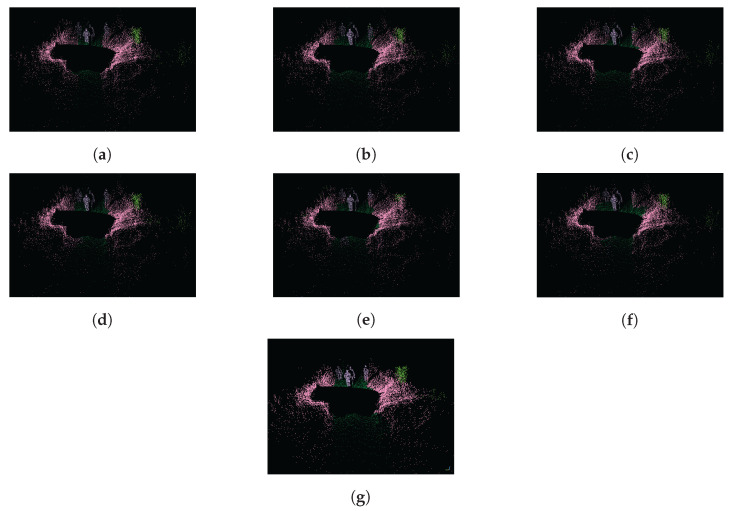
Semantic segmentation results of the RELLIS-3D dataset are presented as point clouds. (**a**) Ground Truth; (**b**) SegUNet3D; (**c**) SegNet; (**d**) U-Net; (**e**) SqueezeSegV2; (**f**) PointSeg; (**g**) SalsaNext.

The comparative results of the RELLIS-3D dataset are presented in Table 8. The ensemble SegUNet3D method outperforms SegNet and U-Net according to the mIoU metric. Compared to image-based methods, SegUNet3D has higher IoU for small and regular objects such as the pole, vehicle, and barrier. Labels that SegNet and U-Net individually assign incorrectly can be determined correctly with the SegUNet3D architecture, which is a combination of two algorithms. SegUNet3D is also superior to SqueezeSegV2, PointSeg and SalsaNext. SqueezeSegV2, PointSeg, and SalsaNext achieved 28.4%, 27.9%, and 31.3% mIoU, respectively, while SegUNet3D achieved 33.3% mIoU. SegUNet3D improves mIoU about 5% compared to SqueezeSegV2 and PointSeg, and 2% compared to SalsaNext in RELLIS-3D dataset. SegUNet3D can successfully extract some small objects that other range image-based methods hardly recognize, such as pole and barrier. The semantic segmentation results of RELLIS-3D dataset are shown in Figure 8 and Figure 9.

Semantic perception of the environment has an important task for successful autonomous driving. According to the results obtained, the SegUNet3D algorithm offers a solution for semantic perception in autonomous driving in areas with different topographic structures. Especially by including 3D geometric features, mIoU values have been increased and a better semantic segmentation performance has been provided. SegUNet3D algorithm can also be used for real-time object detection and navigation. Scene evaluation rates of SegUNet3D are 28.6 images/s for 64 × 512 pixel image, 17.6 images/s for 64 × 1024 pixel image and 7.4 images/s for 64× 2048 pixel image, respectively. SegUNet3D proposes an efficient, fast, and highly accurate solution for semantic segmentation for mobile LiDAR point clouds.

## 5. Conclusions

In this study, we proposed a projection-based deep learning approach, named SegUNet3D, for semantic segmentation of mobile point clouds. The proposed method has been compared with three projection-based and two image-based methods using two public challenge mobile LiDAR datasets. It was demonstrated that it provides better segmentation accuracy. Mobile point clouds are often used for models of dynamic scenes. Moving objects such as people, vehicles, or other living beings in the environment can cause noise. Future studies will focus on eliminating noise caused by moving objects and producing HD maps from mobile point clouds. Additionally, studies on point cloud and image integration can be carried out for the usage of not only geometric features but also radiometric features for point cloud segmentation.

## Figures and Tables

**Table 1:** Results of different network input size on SemanticPOSS. The values are in %.

Resolution	U-Net	SegNet	SqueezeSegV2	PointSeg	SalsaNext	SegUnet3D
64 × 512	29.1	34.4	25.9	25.5	21.8	35.2
64 × 1024	35.6	41.6	35.5	28.8	43.8	44.7
64 × 2048	42.7	45.0	42.4	33.2	42.0	45.7

**Table 2:** Results of different network input size on RELLIS-3D. The values are in %.

Resolution	U-Net	SegNet	SqueezeSegV2	PointSeg	SalsaNext	SegUnet3D
64 × 512	18.5	28.5	26.5	26.0	31.3	33.3
64 × 1024	23.9	29.9	29.1	29.6	32.0	30.6
64 × 2048	28.8	28.7	26.3	24.8	31.6	29.8

**Table 3:** Results of different segment size on SemanticPOSS. The values are in %.

Minimum Points	U-Net	SegNet	SqueezeSegV2	PointSeg	SalsaNext	SegUnet3D
30	35.4	40.7	35.9	29.7	38.3	42.3
50	35.6	41.6	35.5	28.8	43.8	44.7
70	33.4	41.8	35.8	30.1	39.6	41.6

**Table 4:** Results of different segment size on RELLIS-3D. The values are in %.

Minimum Points	U-Net	SegNet	SqueezeSegV2	PointSeg	SalsaNext	SegUnet3D
30	32.9	28.8	27.8	26.7	31.0	30.0
50	18.5	28.5	26.5	26.0	31.3	33.3
70	30.9	29.5	28.4	27.9	25.2	30.3

**Table 5:** The results of usage of 3D geometric features on SemanticPOSS. The values are in %.

Feature	U-Net	SegNet	SqueezeSegV2	PointSeg	SalsaNext	SegUnet3D
Without 3D Geometric Features	38.1	38.6	33.3	28.5	38.6	41.4
With 3D Geometric Features	35.6	41.6	35.5	28.8	43.8	44.7

**Table 6:** The results of usage of 3D geometric features on RELLIS-3D. The values are in %.

Feature	U-Net	SegNet	SqueezeSegV2	PointSeg	SalsaNext	SegUnet3D
Without 3D Geometric Features	32.4	24.4	24.0	24.5	24.9	26.6
With 3D Geometric Features	18.5	28.5	26.5	26.0	31.3	33.3

**Table 7:** Evaluation results on the SemanticPOSS dataset. The values are in %. The highest mIoU value is shown as bold.

Method	People	Rider	Car	Trunk	Plants	Traffic Sign	Pole	Building	Fence	Bike	Road	mIoU
SegNet [34]	42.6	14.8	50.3	24.5	68.2	22.3	12.4	65.9	37.0	43.6	75.7	41.6
U-Net [32]	37.5	1.4	42.1	23.2	62.7	16.4	9.6	62.4	24.3	37.4	74.9	35.6
SqueezeSegV2 [1]	28.3	2.2	42.3	13.3	67.0	13.0	10.4	63.1	32.3	40.8	77.7	35.5
PointSeg [23]	22.5	4.7	21.9	15.1	55.9	13.0	10.0	54.1	17.5	30.0	72.3	28.8
SalsaNext [26]	47.7	6.2	47.1	24.6	69.3	29.3	19.1	64.9	46.9	49.0	78.1	43.8
SegUnet3D (Ours)	44.7	26.4	50.7	24.2	69.2	21.9	17.3	68.4	45.8	46.5	76.3	**44.7**

**Table 8:** Evaluation results on the RELLIS-3D dataset. The values are in %. The highest mIoU value is shown as bold.

Method	Grass	Tree	Pole	Water	Vehicle	Log	Person	Fence	Bush	Concrete	Barrirer	Puddle	Mud	Rubble	mIoU
SegNet [34]	67.3	74.4	0.0	0.0	4.7	0.0	78.1	0.5	75.3	58.7	43.1	2.8	2.9	0.6	29.2
U-Net [32]	67.6	76.4	1.4	20.0	7.6	0.1	77.5	0.6	76.3	62.0	40.6	2.5	2.1	8.5	30.2
SqueezeSegV2 [1]	66.7	73.0	0.0	0.0	3.0	0.0	71.8	0.0	73.4	62.7	39.4	2.7	3.7	0.4	28.4
PointSeg [23]	64.1	67.2	16.0	0.0	12.3	1.1	61.3	6.0	72.2	54.9	24.9	4.4	6.6	0.0	27.9
SalsaNext [26]	67.3	75.5	0.0	0.0	4.1	0.0	82.6	0.2	75.3	66.8	53.3	3.8	3.8	4.7	31.3
SegUnet3D (Ours)	67.6	73.9	39.8	0.0	9.3	0.0	77.5	1.1	75.5	62.2	50.5	3.1	4.7	1.4	**33.3**

## Data Availability

Not applicable.

## References

[1] Wu B., Zhou X., Zhao S., Yue X., Keutzer K. SqueezeSegV2: Improved model structure and unsupervised domain adaptation for road-object segmentation from a LiDAR point cloud. Proceedings of the 2019 International Conference on Robotics and Automation (ICRA).

[2] Biasutti P., Lepetit V., Aujol J.F., Bredif M., Bugeau A. LU-net: An efficient network for 3D LiDAR point cloud semantic segmentation based on end-to-end-learned 3D features and U-net. Proceedings of the IEEE/CVF International Conference on Computer Vision Workshops.

[3] Li S., Liu Y., Gall J. (2021). Rethinking 3-D LiDAR Point Cloud Segmentation. IEEE Trans. Neural Netw. Learn. Syst..

[4] Li Y., Ma L., Zhong Z., Liu F., Chapman M.A., Cao D., Li J. (2021). Deep Learning for LiDAR Point Clouds in Autonomous Driving: A Review. IEEE Trans. Neural Netw. Learn. Syst..

[5] Nagy B., Benedek C. (2019). 3D CNN-based semantic labeling approach for mobile laser scanning data. IEEE Sens. J..

[6] Atik M.E., Duran Z., Seker D.Z. (2021). Machine learning-based supervised classification of point clouds using multiscale geometric features. ISPRS Int. J. Geo-Inf..

[7] Atik M.E., Duran Z. (2021). Classification of Aerial Photogrammetric Point Cloud Using Recurrent Neural Networks. Fresenius Environ. Bull..

[8] Qi C.R., Su H., Mo K., Guibas L.J. PointNet: Deep learning on point sets for 3D classification and segmentation. Proceedings of the IEEE Conference on Computer Vision and Pattern Recognition.

[9] Griffiths D., Boehm J. (2019). A Review on deep learning techniques for 3D sensed data classification. Remote Sens..

[10] Qi C.R., Yi L., Su H., Guibas L.J. PointNet++: Deep hierarchical feature learning on point sets in a metric space. Proceedings of the Advances in Neural Information Processing Systems.

[11] Engelmann F., Kontogianni T., Schult J., Leibe B. Know what your neighbors do: 3D semantic segmentation of point clouds. Proceedings of the European Conference on Computer Vision (ECCV) Workshops.

[12] Hu Q., Yang B., Xie L., Rosa S., Guo Y., Wang Z., Trigoni N., Markham A. Randla-Net: Efficient semantic segmentation of large-scale point clouds. Proceedings of the IEEE/CVF Conference on Computer Vision and Pattern Recognition.

[13] Li Y., Bu R., Sun M., Wu W., Di X., Chen B. PointCNN: Convolution on X-transformed points. Proceedings of the Advances in Neural Information Processing Systems.

[14] Zhao H., Jiang L., Fu C.W., Jia J. Pointweb: Enhancing local neighborhood features for point cloud processing. Proceedings of the IEEE/CVF Conference on Computer Vision and Pattern Recognition.

[15] Zhang Z., Hua B.S., Yeung S.K. ShellNet: Efficient point cloud convolutional neural networks using concentric shells statistics. Proceedings of the IEEE/CVF International Conference on Computer Vision.

[16] Thomas H., Qi C.R., Deschaud J.E., Marcotegui B., Goulette F., Guibas L. KPConv: Flexible and deformable convolution for point clouds. Proceedings of the IEEE/CVF International Conference on Computer Vision.

[17] Wen C., Yang L., Li X., Peng L., Chi T. (2020). Directionally constrained fully convolutional neural network for airborne LiDAR point cloud classification. ISPRS J. Photogramm. Remote Sens..

[18] Yousefhussien M., Kelbe D.J., Ientilucci E.J., Salvaggio C. (2018). A multi-scale fully convolutional network for semantic labeling of 3D point clouds. ISPRS J. Photogramm. Remote Sens..

[19] Maturana D., Scherer S. VoxNet: A 3D Convolutional Neural Network for real-time object recognition. Proceedings of the 2015 IEEE/RSJ International Conference on Intelligent Robots and Systems (IROS).

[20] Zhou Y., Tuzel O. VoxelNet: End-to-End Learning for Point Cloud Based 3D Object Detection. Proceedings of the IEEE Conference on Computer Vision and Pattern Recognition, Salt Lake City.

[21] Wu B., Wan A., Yue X., Keutzer K. SqueezeSeg: Convolutional Neural Nets with Recurrent CRF for Real-Time Road-Object Segmentation from 3D LiDAR Point Cloud. Proceedings of the 2018 IEEE International Conference on Robotics and Automation (ICRA).

[22] Iandola F.N., Han S., Moskewicz M.W., Ashraf K., Dally W.J., Keutzer K. (2016). Squeezenet: AlexNet-level accuracy with 50x fewer parameters and <0.5 MB model size. arXiv.

[23] Wang Y., Shi T., Yun P., Tai L., Liu M. (2018). Pointseg: Real-time semantic segmentation based on 3d lidar point cloud. arXiv.

[24] Milioto A., Vizzo I., Behley J., Stachniss C. RangeNet ++: Fast and Accurate LiDAR Semantic Segmentation. Proceedings of the 2019 IEEE/RSJ international conference on intelligent robots and systems (IROS).

[25] Redmon J., Farhadi A. (2018). Yolov3: An incremental improvement. arXiv.

[26] Cortinhal T., Tzelepis G., Aksoy E.E. SalsaNext: Fast, Uncertainty-Aware Semantic Segmentation of LiDAR Point Clouds. Proceedings of the International Symposium on Visual Computing.

[27] Jiang P., Osteen P., Wigness M., Saripalli S. RELLIS-3D Dataset: Data, Benchmarks and Analysis. Proceedings of the IEEE International Conference on Robotics and Automation (ICRA).

[28] Pan Y., Gao B., Mei J., Geng S., Li C., Zhao H. SemanticPOSS: A Point Cloud Dataset with Large Quantity of Dynamic Instances. Proceedings of the 2020 IEEE Intelligent Vehicles Symposium (IV).

[29] Duran Z., Ozcan K., Atik M.E. (2021). Classification of Photogrammetric and Airborne LiDAR Point Clouds Using Machine Learning Algorithms. Drones.

[30] West K.F., Webb B.N., Lersch J.R., Pothier S., Triscari J.M., Iverson A.E. Context-driven automated target detection in 3D data. Proceedings of the Automatic Target Recognition XIV.

[31] Weinmann M., Jutzi B., Hinz S., Mallet C. (2015). Semantic point cloud interpretation based on optimal neighborhoods, relevant features and efficient classifiers. ISPRS J. Photogramm. Remote Sens..

[32] Ronneberger O., Fischer P., Brox T. U-net: Convolutional networks for biomedical image segmentation. Proceedings of the International Conference on Medical Image Computing and Computer-Assisted Intervention.

[33] Atik S.O., Ipbuker C. (2021). Integrating convolutional neural network and multiresolution segmentation for land cover and land use mapping using satellite imagery. Appl. Sci..

[34] Badrinarayanan V., Kendall A., Cipolla R. (2017). SegNet: A Deep Convolutional Encoder-Decoder Architecture for Image Segmentation. IEEE Trans. Pattern Anal. Mach. Intell..

